# Tailoring Immunization Programmes: using patient file data to explore vaccination uptake and associated factors

**DOI:** 10.1080/21645515.2020.1769396

**Published:** 2020-06-23

**Authors:** Sanjin Musa, Katrine Bach Habersaat, Cath Jackson, Aida Kulo, Emilija Primorac, Mirsad Smjecanin, Sebastian Funk

**Affiliations:** a Department of Epidemiology, Institute for Public Health of the Federation of Bosnia and Herzegovina, Sarajevo, Bosnia and Herzegovina; b Vaccine-preventable Diseases and Immunization, World Health Organization Regional Office for Europe, Copenhagen, OE, Denmark; c Valid Research Limited, West Yorkshire, UK; d Institute of Pharmacology, Clinical Pharmacology and Toxicology, Medical Faculty University of Sarajevo, Sarajevo, Bosnia and Herzegovina; e Department of Infectious Disease Epidemiology, Faculty of Epidemiology and Public Health, London School of Hygiene & Tropical Medicine, London, UK; f Center for the Mathematical Modelling of Infectious Diseases, London School of Hygiene & Tropical Medicine, London, UK

**Keywords:** Vaccination, immunization, immunization, bosnia and herzegovina, tailoring immunization programmes (TIP), parent, determinant

## Abstract

Vaccination uptake in the Federation of Bosnia and Herzegovina (FBiH), in Bosnia and Herzegovina, is suboptimal. This study aimed to (1) assess vaccination coverage, timeliness and drop-out for children born in 2015 and 2016 and compare these with official administrative coverage estimates, (2) identify associations between characteristics of children/caregivers and vaccination uptake. This was a cross-sectional study based on patient files for children 12–23 months (n = 1800) and 24–35 months (n = 1800). Methods were adapted from the World Health Organization cluster survey methodology. A two-stage stratified sampling procedure was conducted in urban and rural strata. A structured paper-based form was completed by a pediatrician/nurse from randomly selected primary care centers and patient files. Estimates were based on weighted analysis with a 95% confidence interval to account for the survey sampling design. Vaccination coverage was consistent with administrative coverage levels for BCG, DTP and MMR, and lower for HepB; all considerably lower than regional targets. Children in urban areas had lower vaccination uptake. An assumption that anti-vaccination sentiment prevails among caregivers was not confirmed; only 2% of children were not vaccinated at all, instead challenges related to delays and drop-out. An assumption of caregiver concerns for the MMR vaccine was confirmed with low uptake and delays. The FBiH has experienced vaccination schedule changes due to supply issues; findings confirmed that sustainability in supply and schedule is high priority. These data are new and provide important information for developing strategies to increase uptake.

## Introduction

Vaccination saves lives and prevents suffering for millions of people every year,^1^ yet vaccination uptake at national and sub-national levels across the World Health Organization (WHO) European Region remains insufficient to stop the spread of measles and other potentially life-threatening vaccine-preventable diseases.^2^ The Federation of Bosnia and Herzegovina (FBiH) is one of the two entities comprising Bosnia and Herzegovina. Vaccination uptake in the FBiH has been declining in recent years. According to administrative official data, coverage for three doses of diphtheria-tetanus-pertussis (DTP) and polio vaccine in children under 1 year declined from 86.2% to 72.3% during 2014–2018; while uptake of the first dose of measles-mumps-rubella vaccine (MMR) declined from 89.1% to 68.4%,^3^ far from the 95% target set for both vaccines in the European Vaccine Action Plan.^4^

A measles outbreak in the FBiH in 2014–2015 reached 5084 cases,^5^ and a current measles outbreak has seen 1332 cases during 2019, with two related deaths of children under 1 year. In its most recent report, the European Regional Certification Commission for Poliomyelitis Eradication concluded that the FBiH, due to its low population immunity, remains at high risk of sustained polio outbreak following importation.^6^

There are no evidence-based data on the reasons for the suboptimal vaccination uptake in the FBiH to inform the development of tailored public health interventions. Some frequent assumptions amongst key stakeholders include caregiver and health worker vaccine hesitancy, misinformation, lack of trust, health worker shortage, vaccine shortages, and immunization schedule changes. Moreover, a comprehensive analysis has not been done to describe vaccinated and unvaccinated population groups in terms of characteristics such as birth order, residence, education, age or community affiliations. Lastly, there are concerns as regards the denominators used to calculate administrative coverage due to inaccurate projections and thus the accuracy of assessment. Administrative coverage is assessed by routine reporting, starting from a health-care facility that performs mandatory immunization, which is compiled by cantonal public health institutes and passed to the Institute for Public Health of FBiH. The number of doses administered to the target population is divided by the total estimated number of people in the target population.

Given these challenges, the Institute for Public Health of the FBiH decided to initiate a WHO Tailoring Immunization Programmes (TIP) project to understand barriers and drivers to childhood vaccination, to inform a strategy to increase vaccination uptake.^7–9^ The TIP approach applies a people-centered approach and draws on the COM-B (capability, opportunity, motivation-behavior) system as a framework for changing behavior.^10^ Three TIP formative research studies were conducted in the FBiH from 2017 to 2019: two qualitative interview studies with health workers and caregivers,^11^ to identify barriers and drivers to positive vaccination behaviors, and the patient file study presented in this paper. The aims of this study were to:

1. assess the vaccination coverage, timeliness and drop-out for children born in 2015 and 2016; and compare these with official administrative coverage estimates;
2. identify associations between the characteristics of children/caregivers and vaccination uptake.

## Methods

The childhood schedule for the FBiH is presented in Table 1. Throughout the paper, we refer to vaccines by their abbreviation with a number to indicate the dose (e.g. DTP3 is the third dose of diptheria-tetanus-pertussis-containing vaccine).

**Table 1.** Childhood immunization schedule in FBiH in 2016

Vaccine	Age
Bacillus Calmette Guérin (BCG)	Neonates
Hepatitis B (HepB)	Neonates, 1, 6 months
Diphtheria-tetanus-acellular pertussis (DTaP)^1^ Inactivated polio vaccine (IPV) ***Haemophilus*** influenzae type b (Hib)	2, 4, 6 months
Measles-mumps-rubella (MMR)	12 months
Oral polio vaccine (OPV)-booster	18 months
Hib-booster (for children who received monovalent vaccine)	18 months
DTaP-IPV booster	4 years
MMR	5 years
Diphtheria-tetanus (dT) OPV-booster	13–14 years
Tetanus-booster	17–18 years

^1^From 2014 to 2016 there was a global shortage of combined vaccines with acellular pertussis. In 2015, the tetravalent (DTaP-IPV + Hib) vaccine (diphtheria, tetanus, acellular pertussis, and polio given simultaneously with haemophilus influenzae type b) used in the primary series was replaced with trivalent (DTwP + OPV + Hib) vaccine (diphtheria, tetanus, whole-cell pertussis, given simultaneously with oral polio and haemophilus influenzae type b). Only the first dose of the tetravalent vaccine remained in the schedule. By mid-2016, pentavalent (DTaP-IPV-Hib) vaccine (diphtheria, tetanus, acellular pertussis, polio and haemophilus influenzae type b) was introduced into the schedule for the first time. In 2017, the third dose of pentavalent vaccine was moved from 6 to 10 months, and monovalent Hib-booster was removed from the schedule, together with third OPV-booster and tetanus-booster.

### Study design and target population

This was a cross-sectional study, based on Primary Health Care (PHC) facility patient files of children, which included data about their caregivers.

We included two cohorts of children, born in 2015 (aged 24–35 months at the time of data analysis) and 2016 (aged 12–23 months at the time of data analysis) because of the change in pertussis vaccine that occurred at this time (see Table 1 footnote). Children born in 2015 temporarily received a vaccine containing whole-cell pertussis (not acellular) which has been the issue of dispute in the past;^12^ thus, to minimize bias related to this change in the schedule and the fact that it also affected HepB3 vaccine coverage, which is given simultaneously, we only reviewed MMR uptake for these children, enabling us to review MMR1 uptake beyond 12 months of age. Children born in 2016 mainly received acellular pertussis-containing vaccine; we, therefore, included all vaccines except MMR1 in studying this cohort. The inclusion of this cohort enabled us to review uptake in vaccinations that are scheduled in the first 12 months of life.

### Operational definitions

#### Fully vaccinated

- Child aged 12–23 months who received BCG, DTP3, HepB3
- Child aged 24–35 months who received MMR1

#### Unvaccinated

- Child aged 12–23 months who had not received any of BCG, DTP or HepB. To account for the fact that many children receive their first doses of two vaccinations (BCG and HepB1) in birth clinics, we also investigated children who were unvaccinated except for those first doses.
- Child aged 24–35 months who had not received MMR1.

#### Timeliness

- Child who had received vaccines on time according to the childhood schedule for the FBiH

#### Drop-out

- Child who had received the first dose, but not the third dose of DTP or HepB:
  - DTP drop-out: (DTP1 – DTP3/DTP1) * 100
  - HepB drop-out: (HepB1 – HepB3/HepB1) * 100

### Sample size and sampling technique

The sampling frame and procedures were adapted from the WHO Expanded Programme on Immunization (EPI) cluster survey.^13^

The sample size was calculated to allow us to estimate the FBiH coverage with a confidence level no wider than 8%, when coverage is between 50% and 70%, and with a design effect of 5.67. Estimated total target participants: 2 (strata – urban/rural) x 159 (effective sample size) x 5.67 (design effect) = 1803. The final sample size was rounded down to 1800 participants per age group (900 for each urban and rural strata). The number of patient files to check per cluster was 15, based on the number of households a data collection team can visit in a day as well as the total number of target respondents expected in an average size cluster, assuming that all eligible respondents in those households visited are interviewed. Since the sample size selection was done in the PHC facility (not in the community), we defined a cluster as a unit of 60 (4x15) patient files.

To categorize settlements as rural/urban, a 2013 census list of FBiH municipalities by settlement type (rural/urban) was used. Municipalities with at least 65% urban households were classified as urban.^14^

Seventy-nine PHCs in the FBiH provide childhood vaccination. The sizes of the birth cohorts were 19,358 in 2015 and 19,655 in 2016.^15,16^

A two-stage stratified sampling procedure was conducted in each stratum (urban/rural). A sampling frame was created by listing all the PHCs alongside the number of children in the birth cohort from each PHC and then further segmenting the PHCs into clusters (primary sampling units) comprising 60 patient files. PHCs with fewer than 60 patient files were combined with neighboring PHCs to create a single cluster. Next, we randomly selected 15 clusters in each stratum using Microsoft Excel with the formula for randomization. Since the sampling units had been split into sub-units (clusters), the weight template was adapted. We computed a new measure of size by dividing the sum of patient files in a PHC (or collection of PHCs) by the resultant number of units created. All selected PHCs agreed to participate and received a small fee for their participation.

Within the clusters selected in the first stage of the sampling procedure, we randomly selected patient files for children born in 2015 and 2016, using Microsoft Excel with the formula for randomization. Patient files from a total of 3600 (1800 children per age group) were reviewed during January 2018.

Normalized and post-stratified weights were computed. The weights were normalized to the total number of selected units; hence, each weight was multiplied by the sum of samples divided by the sum of weighted number of children (sum of individual weights multiplied by the sample).

$$f_{i}\left(x\right)=\frac{w^{x_{i}}\times S^{x_{i}}}{\sum_{j}w^{x_{i}}.S^{x_{i}}}$$

The normalized weights within a stratum were multiplied by a factor that limits the sum of these weights to the proportionate contribution of each strata in the sampling frame (Federation). All estimates presented in the paper are based on weighted analysis to account for the survey sampling design.

### Data collection

A structured paper-based form was developed, pre-tested and completed by a pediatrician/nurse from the selected PHCs. A sub-sample was overseen by trained supervisors (MS, EP). Where data were missing the pediatrician/nurse telephoned caregivers. With no official classification of ethnicity, categorization of Roma/other was based on the knowledge of the pediatrician/nurse.

Characteristics recorded in the form were:

- Child: gender, birth order, residence (urban/rural), community affiliation (Roma/other)
- Caregiver: education (none/low/medium/high), age, number of children

Data were entered into MS Access 2013.

### Data analysis

Descriptive statistics were generated to characterize the sample and weighted analyses to calculate coverage rates with a 95% confidence interval (CI) for each vaccine/dose and for the measurements of a fully vaccinated child. Bivariate and multivariate analyses were done to explore the association between each of the possible explanatory variables and the outcome variable of fully vaccinated. Chi-square tests were used to compare levels of uptake, and cox proportional hazard regression for comparing timeliness. Analyses were conducted using the R package *srvyr*.

### Ethical approval

Ethical approval was secured from the Ethics Committee of the Institute for Public Health of the FBiH. Only authorized supervisors and pediatricians/nurses who already had access to patient files reviewed these. Data were recorded anonymously, no names were kept in the study database.

## Results

### Child and caregiver characteristics

The characteristics of the 1800 children and their caregivers in both cohorts are presented in Table 2. Both weighted (by population size) and non-weighted data are presented because the urban and rural classification did not represent an equal proportion of the population once weighted. For both age groups just over half of the sample was boys; and the majority was a first or second born child. Most mothers were aged 24–35 years and most fathers were 28–37 years old. In terms of education, medium level (high school) was dominant among mothers and fathers. Twenty children aged 12–23 months and 16 children aged 24–35 months were identified as Roma.

**Table 2.** Characteristics of children and their caregivers by birth cohort 2016 and 2015 (unweighted and weighted), FBiH January 2018

Characteristic	Children born in 2016 (aged 12–23 months)		Children born in 2015 (aged 24–35 months)	
	Unweighted n	Weighted n (95% CI)	Unweighted n	Weighted n (95% CI)
Total	1800	1800 (1658–1942)	1800	1800 (1658–1942)
Gender of child				
*Male*	920	927 (821–1034)	898	894 (816–971)
*Female*	874	867 (792–943)	878	877 (795–960)
Birth order^1^				
*1*	762	791 (647–934)	684	681 (541–821)
*2*	621	609 (564–654)	608	609 (513–705)
*3*	168	170 (111–229)	184	189 (139–239)
*>3*	45	47 (25–69)	45	56 (11–100)
Residence				
*Urban*	900	674 (668–681)	900	674 (668–681)
*Rural*	900	1126 (983–1268)	900	1126 (983–1268)
Mother’s education^2^				
*None*	5	5 (0–10)	8	9 (0–18)
*Low*	83	87 (53–120)	70	71 (40–103)
*Medium*	1044	1080 (938–1223)	991	1020 (859–1181)
*High*	591	547 (455–638)	570	529 (430–627)
Father’s education^2^				
*None*	5	5 (0–10)	7	8 (0–15)
*Low*	56	57 (35–80)	36	39 (18–59)
*Medium*	1230	1265 (1123–1407)	1203	1219 (1038–1399)
*High*	440	400 (342–457)	404	375 (308–442)
Mother’s age (years)^3^				
*<24*	200	207 (156–259)	115	116 (79–153)
*24–29*	526	548 (469–627)	462	490 (385–596)
*30–35*	674	665 (564–766)	690	677 (571–784)
*>35*	288	275 (214–337)	345	331 (265–396)
Father’s age (years)^3^				
*<28*	228	236 (191–281)	144	146 (115–176)
*28–32*	642	645 (560–729)	599	599 (494–705)
*33–37*	528	536 (447–624)	580	577 (490–664)
*>37*	283	272 (219–325)	293	294 (221–367)
Community affiliation				
*Other*	1780	1780 (1634–1926)	1784	1782 (1638–1925)
*Roma*	20	20 (5–35)	16	18 (7–30)

^1^Birth order was defined as 1 (first), 2 (second), 3 (third), >3 (forth or later child); ^2^Mother’s/Father’s education was defined as low (primary school), medium (high school), high (university degree); ^3^Male/female age groups were chosen to ensure even spread across groups. Average age of mothers was 31.7 (31.2–32.3) years and fathers 35.1 (34.6–35.6) years.

### Vaccination coverage

Vaccination coverage data by residence among children born in 2015 and 2016 are presented in Table 3.

**Table 3.** Vaccination coverage by residence among children born in 2016 and 2015, FBiH January 2018

Vaccine	Total % (95% CI)	Urban % (95% CI)	Rural % (95% CI)	P-value (urban/rural)
BCG	98 (97–99)	97 (95–98)	99 (98–100)	0.059
DTP1-containing vaccine	86 (81–90)	79 (73–84)	90 (84–96)	0.014
DTP2-containing vaccine	78 (72–84)	70 (63–76)	83 (74–91)	0.029
DTP3-containing vaccine	64 (55–72)	52 (44–59)	71 (59–83)	0.017
HepB1	98 (96–99)	96 (95–98)	99 (97–100)	0.13
HepB2	90 (86–94)	86 (82–90)	93 (87–98)	0.088
HepB3	64 (57–71)	51 (43–59)	72 (61–82)	<0.01
MMR1^1^	65 (60–71)	51 (44–57)	74 (66–82)	<0.01

^1^Data for children aged 24–35 months only. *Note. P*-values for the difference between urban and rural coverage were calculated using a chi-squared test.

Coverage was lower for all vaccines for urban residences compared to rural. The differences were most pronounced for the three doses of DTP-containing vaccine, HepB3 and MMR1. The vaccines administered at birth, BCG and HepB1 had the highest coverage (BCG: 98%, 95% CI 97–99%; HepB1: 98%, 95% CI 96–99%). Notably, HepB coverage decreased with each dose, leading to coverage for the third dose of 64% (95% CI 57–71%). The same pattern was evident for DTP-containing vaccine with coverage decreasing from 86% (95% CI 81–90%) for first dose to 64% (95% CI 55–72%) at third dose. Among children aged 24–35 months, MMR1 coverage was 65% (95% CI 60–71%).

Just 2% (95% CI 1–3%) had received no vaccinations at all (Table 3). To account for the fact that many children routinely receive their first dose of vaccinations at birth, we also looked at children who were unvaccinated except for BCG and HepB1. A total of 8% (95% CI 4–11%) was unvaccinated using this definition.

**Figure 1.**
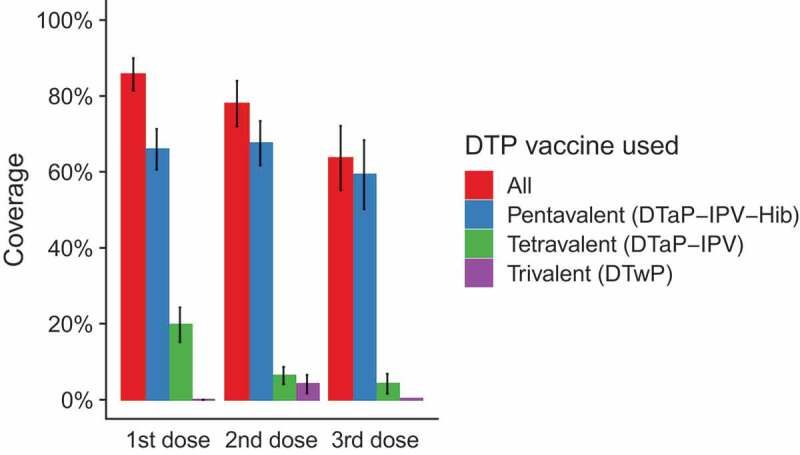
DTP vaccination coverage in 2016, by vaccines used, FBiH January 2018

For DTP, pentavalent vaccine was the most commonly administered (coverage with 1st dose 66%, 2nd dose 68%, 3rd dose 59%), and trivalent vaccine was the least frequently administered (coverage with 1st dose 0%, 2nd dose 4%. 3rd dose 0%). The distribution of vaccines used for the first, second and third dose is presented in Figure 1.

#### Patient file data versus official administrative coverage estimates

**Figure 2.**
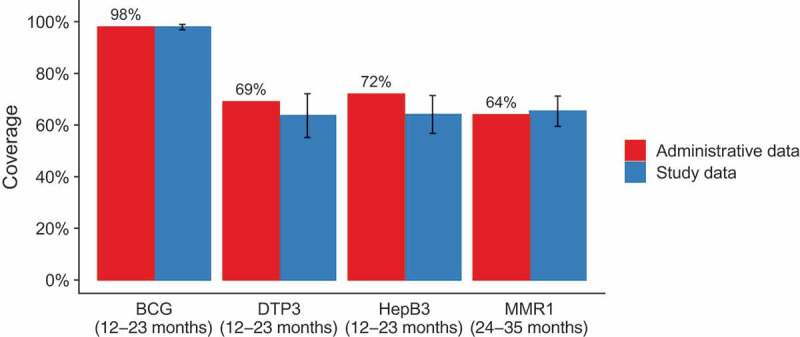
Vaccination coverage: patient file data compared with official administrative, FBiH January 2018^3^ *Note*. Error bar represents one 95% CI. The coverage levels estimated from our study data are shown in Table 3

Vaccination coverage data from the patient files were compared with the data reported in the official annual report (Figure 2). Coverage levels estimated in our study were consistent with administrative levels for three of the four vaccines (*p*-values for null hypothesis of no difference: BCG 0.93, DTP3 0.21, MMR1 0.63), but to a lesser degree for HepB3 coverage, which was estimated to be lower than the administrative level (64% vs. 72%, *p* = .04).

#### Timeliness

To assess timeliness, the age of children at the time of vaccination was assessed for HepB, DTP, and MMR1.

**Figure 3.**
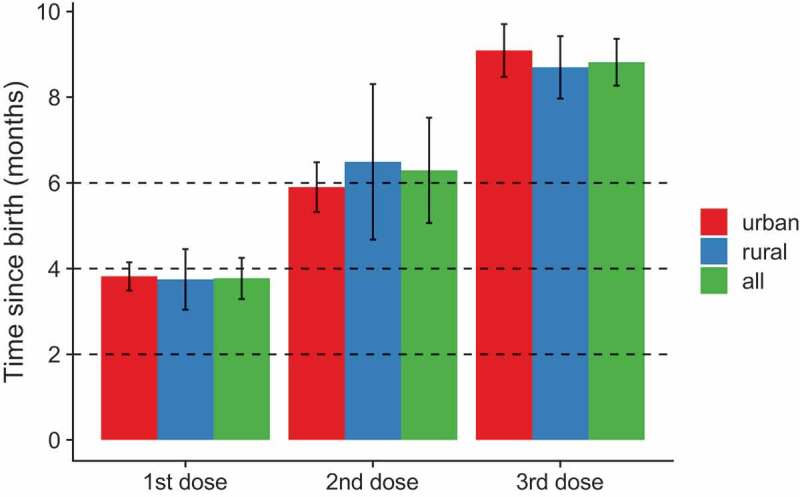
Timing of vaccination by residence for DTP-containing vaccine, children born in 2016 and aged 12–23 months, FBiH January 2018

*Note*. Error bar represents one 95% CI. Dotted line represents the recommended timing of vaccination.

**Figure 4.**
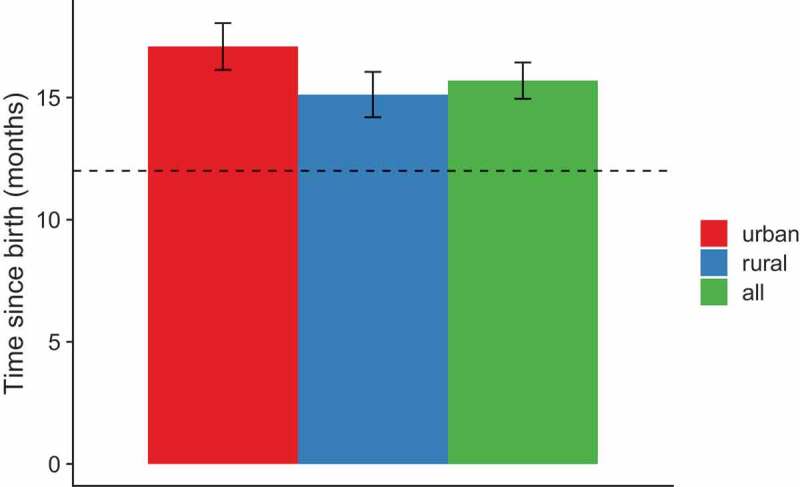
Timing of vaccination by residence for MMR1, children born in 2015 and aged 24–35 months, FBiH January 2018

*Note*. Error bar represents one 95% CI. Dotted line represents the recommended timing of vaccination.

The recommended timing for the three doses of HepB was at birth, 1 and 6 months (or at least 6 months after the first dose) and for DTP at 2, 4 and 6 months (Table 1). For children who received all three doses of each vaccine, the timing of vaccination by residence for each dose is presented in Table 4. Mean age for all three doses was older than the recommended age for both vaccines, with completion of each vaccine at 8–9 months instead of the recommended 6 months. Vaccination occurred later in urban than rural settings for MMR1, and a slightly significant difference was found for HepB1 (Table 4, Figures 3 and 4). Mean age for DTP1 was 3.8 months.

**Table 4.** Time of vaccination in months by residence among children born in 2016 and 2015, FBiH January 2018

Vaccine	All mean (95% CI)	Urban mean (95% CI)	Rural mean (95% CI)	P-value (urban/rural)
BCG	0.30 (0.24–0.35)	0.31 (0.22–0.40)	0.29 (0.22–0.36)	0.23
DTP1-containing vaccine	3.8 (3.3–4.3)	3.8 (3.5–4.1)	3.7 (3.0–4.5)	0.88
DTP2-containing vaccine	6.3 (5.1–7.5)	5.9 (5.3–6.5)	6.5 (4.7–8.3)	0.96
DTP3-containing vaccine	8.8 (8.3–9.4)	9.1 (8.5–9.7)	8.7 (8.0–9.4)	0.49
HepB1	0.33 (0.27–0.39)	0.40 (0.29–0.52)	0.29 (0.23–0.36)	0.044
HepB2	1.9 (1.7–2.1)	2 (1.8–2.2)	1.9 (1.6–2.1)	0.38
HepB3	8.8 (8.2–9.4)	9.1 (8.5–9.7)	8.6 (7.8–9.4)	0.43
MMR1^1^	16 (15–16)	17 (16–18)	15 (14–16)	<0.01

^1^Data for children aged 24–35 months only. *Note*. The recommended timing for BCG was at birth, for the three doses of HepB were at birth, 1 and 6 months (or at least 6 months after the first dose), for DTP at 2, 4 and 6 months, and for MMR1 at 12 months. The results for children born in 2016 were assessed for 59% (51–68%) of the whole sample, and for children born in 2015 for 65% (60–71%) of the whole sample. *P*-values for the difference between urban and rural coverage were calculated using a cox proportional hazards model with urban/rural status as sole predictor.

#### Drop out

The drop-out rate for HepB from first to third dose was 34% (95% CI 27–41%). For DTP the rate was 26% (95% CI 19–33%).

### Characteristics associated with full vaccination coverage

Table 5 presents the association between child and caregiver characteristics in relation to full and no vaccination coverage among children born in 2016, aged 12–23 months (excluding MMR). Just over half of the children (59%, 95% CI 51–68%) were fully vaccinated. The odds of being fully vaccinated were three times (95% CI 1.6–5.4) higher for a child living in a rural area compared to an urban area. Conversely, a child of Roma community affiliation was less likely to be fully vaccinated than a non-Roma child (although this is based on a small sample). None of the other child or caregiver characteristics were strongly associated with full vaccination coverage.

**Table 5.** Association between child and caregiver characteristics in relation to BCG, DTP3, HepB3 (full vaccination) coverage among children born in 2016 and aged 12–23 months, FBiH January 2018

Characteristic	Fully vaccinated	Crude odds ratio (95% CI)	Adjusted^1^ odds ratio (95% CI)
Total	59 (51–68)%	n/a	n/a
Gender of child			
*Male*	58 (48–67)%	Baseline	Baseline
*Female*	61 (53–69)%	1.16 (0.94–1.43)	1.09 (0.89–1.33)
Birth order^2^			
*1*	65 (56–74)%	Baseline	Baseline
*2*	57 (48–66)%	0.71 (0.57–0.88)	0.77 (0.60–0.98)
*3*	61 (45–77)%	0.83 (0.49–1.43)	0.87 (0.52–1.46)
*>3*	55 (32–78)%	0.66 (0.29–1.49)	0.80 (0.35–1.85)
Residence			
*Urban*	47 (39–55)%	Baseline	Baseline
*Rural*	67 (55–79)%	2.26 (1.24–4.13)	2.96 (1.63–5.40)
Mother’s education^3^			
*None*	0 (0–0)%	0.00 (0.00–0.00)	0.00 (0.00–0.00)
*Low*	49 (35–63)%	0.60 (0.37–0.97)	0.52 (0.24–1.11)
*Medium*	62 (52–72)%	Baseline	Baseline
*High*	61 (53–68)%	0.97 (0.70–1.34)	1.09 (0.73–1.63)
Father’s education^3^			
*None*	0 (0–0)%	0.00 (0.00–0.00)	1.54 (0.36–6.51)
*Low*	54 (39–69)%	0.73 (0.45–1.19)	1.12 (0.47–2.65)
*Medium*	61 (52–70)%	Baseline	Baseline
*High*	60 (53–67)%	0.95 (0.72–1.25)	1.10 (0.82–1.47)
Mother’s age (years)^4^			
*<24*	58 (43–72)%	0.94 (0.61–1.46)	0.77 (0.45–1.31)
*24–29*	62 (53–71)%	1.12 (0.88–1.44)	0.96 (0.71–1.29)
*30–35*	59 (51–68)%	Baseline	Baseline
*>35*	57 (46–67)%	0.89 (0.71–1.12)	0.95 (0.75–1.20)
Father’s age (years)^4^			
*<28*	60 (47–73)%	Baseline	Baseline
*28–32*	62 (54–70)%	1.10 (0.82–1.47)	0.89 (0.61–1.31)
*33–37*	58 (49–68)%	0.94 (0.65–1.37)	0.76 (0.47–1.21)
*>37*	58 (47–69)%	0.93 (0.61–1.44)	0.86 (0.50–1.50)
Community affiliation			
*Other*	60 (52–68)%	Baseline	Baseline
*Roma*	0 (0–0)%	0.00 (0.00–0.00)	0.00 (0.00–0.00)

^1^All explanatory variables included in the model. ^2^Birth order was defined as 1 (first), 2 (second), 3 (third), >3 (forth or later child); ^3^Mother’s/Father’s education was defined as low (primary school), medium (high school), high (university degree); ^4^Mother/father age groups were chosen to ensure even spread across groups. Average age of mothers was 31.7 (31.2–32.3) years and fathers 35.1 (34.6–35.6) years.

Table 6 presents the association between child and caregiver characteristics in relation to full vaccination coverage for MMR1 among children born in 2015, aged 24–35 months. Two-thirds of the children were fully vaccinated (65%, 95% CI 60–71%). The odds of being vaccinated with MMR1 were 4.7 times (95% CI 2.6–8.2) higher for a child living in a rural area compared to an urban area; and 1.4 times higher (95% CI 1.1–1.8) if the mother is older compared to younger (>35 years to 30–35 years). None of the other child or caregiver characteristics were strongly associated with MMR1 vaccination coverage.

**Table 6.** Association between child and caregiver characteristics in relation to MMR1 (full vaccination) coverage, among children born in 2015 and aged 24–35 months, FBiH January 2018

Characteristic	Fully MMR1 vaccinated	Crude odds ratio (95% CI)	Adjusted^1^ odds ratio (95% CI)
Total	65 (60–71)%	n/a	n/a
Gender of child			
*Male*	64 (58–71)%	Baseline	Baseline
*Female*	67 (60–74)%	1.12 (0.89–1.40)	1.03 (0.78–1.36)
Birth order^2^			
*1*	66 (59–73)%	Baseline	Baseline
*2*	66 (58–74)%	1.00 (0.79–1.27)	0.98 (0.75–1.30)
*3*	69 (62–76)%	1.13 (0.79–1.63)	0.85 (0.56–1.30)
*>3*	71 (57–85)%	1.25 (0.61–2.58)	0.69 (0.34–1.40)
Residence			
*Urban*	51 (44–57)%	Baseline	Baseline
*Rural*	74 (66–82)%	2.81 (1.76–4.49)	4.65 (2.63–8.24)
Mother’s education^3^			
*None*	54 (18–90)%	0.51 (0.13–1.94)	4.26 (0.39–46.02)
*Low*	67 (55–79)%	0.88 (0.52–1.47)	0.96 (0.44–2.06)
*Medium*	70 (64–76)%	Baseline	Baseline
*High*	61 (54–68)%	0.68 (0.53–0.86)	0.83 (0.64–1.07)
Father’s education^3^			
*None*	36 (0–86)%	0.26 (0.03–1.92)	0.11 (0.01–1.51)
*Low*	66 (47–85)%	0.87 (0.42–1.84)	0.71 (0.28–1.77)
*Medium*	69 (64–74)%	Baseline	Baseline
*High*	61 (53–69)%	0.72 (0.57–0.91)	0.89 (0.65–1.22)
Mother’s age (years)^4^			
*<24*	64 (55–74)%	1.02 (0.67–1.54)	0.70 (0.37–1.32)
*24–29*	70 (62–78)%	1.30 (0.95–1.77)	0.95 (0.60–1.51)
*30–35*	64 (58–70)%	Baseline	Baseline
*>35*	68 (62–74)%	1.19 (0.96–1.47)	1.44 (1.13–1.83)
Father’s age (years)^4^			
*<28*	64 (53–74)%	Baseline	Baseline
*28–32*	65 (58–73)%	1.08 (0.72–1.62)	1.28 (0.70–2.34)
*33–37*	68 (62–74)%	1.23 (0.79–1.91)	1.49 (0.75–2.94)
*>37*	67 (60–74)%	1.15 (0.68–1.93)	1.13 (0.53–2.45)
Community affiliation			
*Other*	66 (60–72)%	Baseline	Baseline
*Roma*	22 (0–45)%	0.14 (0.04–0.50)	0.25 (0.03–2.33)

^1^All explanatory variables included in the model. ^2^Birth order was defined as 1 (first), 2 (second), 3 (third), >3 (forth or later child); ^3^Mother’s/Father’s education was defined as low (primary school), medium (high school), high (university degree); ^4^Mother/father age groups were chosen to ensure even spread across groups. Average age of mothers was 31.7 (31.2–32.3) years and fathers 35.1 (34.6–35.6) years.

## Discussion

This study assessed childhood vaccination coverage, timeliness and drop-out in the FBiH and described the characteristics of fully vaccinated children and their caregivers. These data provide detailed insight, and alongside two qualitative TIP studies conducted in parallel,^11^ are important information for developing evidence-informed tailored interventions to improve vaccination coverage in the FBiH.^7–9^

The study confirmed that vaccination coverage in the FBiH in 2016 was considerably lower than regional targets and coverage in other central European countries;^17,18^ and that there are issues related to delay and drop-out for a considerable proportion of children. This increases the risk of outbreaks of vaccine-preventable diseases with personal and financial implications for individuals and health systems. Our findings also confirmed the accuracy of the administrative data.^3^

Prior to conducting this study, there were a number of assumptions in the FBiH about factors related to vaccination of which some were confirmed, and others challenged by the study findings. There was an assumption that anti-vaccination sentiment prevails among caregivers in the FBiH. This notion is challenged by the high coverage for birth doses and vaccines given at 1 and 2 months of age. Indeed, only 2% of the children in this study were completely unvaccinated. Instead, the study identified challenges related to the completion of the vaccination schedule and timeliness. For all assessed vaccines, the study confirmed considerable delays and drop-out. Insight from our qualitative studies with caregivers (unpublished data) and health workers;^11^ and a study in neighboring Serbia,^19^ offer potential explanations for suboptimal vaccination for later doses (delay or drop-out), including changes in immunization schedule, lack of knowledge and/or information about the importance of completing the schedule, lack of encouragement from health workers to return for vaccination; inefficient or a lack of reminders, poorer doctor-patient relationship in urban comparing to rural setting and overloaded pediatricians in urban settings, and competing priorities for caregivers in urban settings.

Another assumption was that caregivers have particular concerns about the MMR vaccine. Indeed, this was evident from our interview studies with caregivers (unpublished) and health workers.^11^ Anti-MMR vaccine information is increasingly circulating in the Balkan countries (sharing similar languages),^20^ and MMR vaccination rates in these countries have declined more significantly than other vaccines.^18^ It is known that some parents delay MMR vaccination due to misperceived risks of potential side effects affecting walking and speaking abilities.^21,22^ This current study confirmed that one-third of eligible children had not received MMR vaccination, higher than for any other vaccines. Specifically, MMR1 vaccine compared to DTP1 vaccine was less accepted (65% versus 86%) and more delayed (4 months versus 2 months).

Another explanation to the general pattern of low coverage at 12 months may be that at the time there were specific concerns to the DTP vaccine. As mentioned, whole-cell pertussis had been temporarily reintroduced. Even if this did not affect the cohort studied to great degree in terms of the *vaccines* which were administered to them, the *debate* related to whole-cell pertussis may have affected both health workers and caregivers. This means that the delays related to DTP vaccine and low uptake of DTP3 may be related to vaccine safety concerns as well.

The study confirmed a considerable delay for vaccines not provided at birth or during initial months. In 2017 (the cohort following our study), due to cost and supply issues, the immunization schedule in the FBiH was changed with DTP3 being postponed from 6 to 10 months. Given the findings of this study, such rescheduling may postpone DTP vaccination even further into the child’s second year. In 2019, thanks to a multi-year tender resulting in a decreased vaccine price and sustainable supply, DTP doses were changed back to 2–4–6 months (including a booster dose in the second year and a pre-school booster dose). The findings of this study indicate that in order to achieve early and durable protection against pertussis disease, the priming schedule with three DTP doses at 2–4–6 months or with a shorter gap between second and third doses, is better optimized compared to a schedule with two doses at 2–4 months and a booster at 10 months of age.

In terms of caregiver/child characteristics, the study clearly showed that children living in urban areas have lower vaccination coverage, evident for all vaccinations except BCG. Differences in the timeliness of vaccination by residence only applied for MMR1 where the delay was more frequent for urban areas. International literature reports a positive impact of maternal education on vaccination coverage,^23^ although conversely a popular assumption, especially with MMR, is that highly educated parents are those most likely to reject vaccination.^24^ Neither were supported by the findings. We found no association with education, and with complete vaccination coverage in urban areas lower than 50%.

Earlier data from the FBiH and wider Europe suggest low vaccination coverage among Roma,^25–27^ and this was confirmed in this study, although this finding should be viewed with caution given the small sample size. Given this finding, additional explanations for suboptimal vaccination based on evidence from other countries could again be speculated and further explored for verification.

Finally, the use of digital technology for the vaccination reporting would ensure accurate coverage estimates, and should be considered for the future activities.

### Strengths and limitations

The study method was unconventional, although based on an accepted WHO methodology.^13^ Probability samples and weighted statistical analyses were used at all stages of sampling, thus reducing the potential for selection bias, and allowing the calculation of meaningful confidence intervals and confidence bounds.^13^

Regular surveys on national vaccination coverage provide important information for public health officials and decision makers, and public health practitioners and researchers are encouraged to conduct them. Typically, evidence is usually collected from home-based records (household surveys), as well as from a vaccination history as recalled by the individual or the child’s caregivers, which can be very expensive and time-consuming. Our approach, using vaccination documentation at the child’s health-care facility, was sufficient to confirm low coverage and provided additional valuable data (which are not included in the administrative data), about timeliness, and the characteristics of children and their caregivers. This method could be adapted and used as a quick and less expensive approach to cluster vaccination coverage survey, if there is documented evidence of vaccination in health facilities. Feedback from participating pediatricians and nurses was that the work was not a significant burden, and they all provided the data within 1 week.

However, this method has several limitations: there might be undocumented vaccination as children may have been vaccinated at multiple health facilities, or in private clinics that were not recorded in the patient file. Although we tried to assess data on evidence for vaccination elsewhere in the files. We found that 1% of children in both age groups had documented vaccination elsewhere. Also, data on caregivers’ characteristics, for example, education and community affiliation were inconsistently registered in files and required additional data collection via phone by the pediatrician/nurse.

Due to vaccine supply issues, coverage was assessed for two cohorts. Broader insights into vaccination coverage and delayed vaccination would have allowed an assessment for a broader age group, for example, up to 5 years; this was not possible within the limitations of this project.

To minimize bias, categories were developed for each question and data collection was supervised by appointed supervisors. As part of quality control, analyses also included internal consistency checks. As there is no official categorization of specific population groups, the categorization of “Roma” vs “other” had to be done based on the knowledge of the person completing the data collection form. At the same time, the Roma part of the sample was small. Findings related to this population group should be interpreted with care and inspire more research.

The findings of this study indicate that comprehensive action, investment and prioritization from decision-makers are immediately necessary for the FBiH to ensure the protection of the population against vaccine-preventable diseases. It is important to ensure a stable supply of mandatory vaccines to avoid vaccine shortages and consequential adjustment of the immunization calendar; to educate health workers and caregivers to improve knowledge related to safety concerns; to improve urban vaccination settings in order to encourage urban caregivers to bring children for vaccination; to support Roma families to bring children for vaccination and finally, and to digitalize the vaccination reporting system to ensure accurate coverage estimates.

## Conclusions

The study confirmed the administrative coverage estimates and that vaccination coverage for children born in 2015 (MMR1) and 2016 (BCG, HepB, DTP) is significantly below the target immunization rates and is associated with considerable delays and drop-out. Very close to no children were not vaccinated at all. The later doses have lower coverage than doses given earlier. This is associated with high drop-out rates and does not confirm a general anti-vaccination sentiment. The study indicated that suboptimal vaccination coverage is associated with: child living in urban area (low coverage and delayed vaccination). Assessment of vaccination coverage of children of caregivers of Roma origin indicated low coverage and needs further exploration.

## Tip advisory group

- Goran Čerkez, Federal Ministry of Health, Federation of Bosnia and Herzegovina, Bosnia and Herzegovina
- Zlatan Peršić, Federal Ministry of Health, Federation of Bosnia and Herzegovina, Bosnia and Herzegovina
- Davor Pehar, Institute for Public Health of the Federation of Bosnia and Herzegovina, Bosnia and Herzegovina
- Mirsada Mulaomerović, Institute for Public Health of the Federation of Bosnia and Herzegovina, Bosnia and Herzegovina
- Victor Olsavszky, WHO Country Office in Bosnia and Herzegovina
- Fatima Čengić, UNICEF Bosnia and Herzegovina
- Amra Junuzović, Health Centre of Sarajevo Canton, Bosnia and Herzegovina
- Jelena Kalinić, Society for Science Advocacy “Science and the World“, Bosnia and Herzegovina

## References

[1] Andre FE, Booy R, Bock HL, Clemens J, Datta SK, John TJ, Lee BW, Lolekha S, Peltola H, Ruff TA, et al. Vaccination greatly reduces disease, disability, death and inequity worldwide. Bull World Health Organ. 2008;86:140–46. doi:10.2471/BLT.07.040089. 18297169 PMC2647387

[2] World Health Organization Regional Office for Europe. WHO Epi Brief: A report on the epidemiology of selected vaccine-preventable diseases in the European Region. Copenhagen: World Health Organization; 2019 [accessed 2019 1126]. http://www.euro.who.int/__data/assets/pdf_file/0017/410714/EpiBrief_2_2019_EN.pdf

[3] Institute for Public Health FBiH. Epidemiological surveillance of communicable diseases in the Federation of Bosnia and Herzegovina. Sarajevo; 2019.

[4] World Health Organization Regional Office for Europe. European Vaccine Action Plan 2015-2020. Copenhagen (Denmark): World Health Organization; 2014 [accessed 2019 530]. http://www.euro.who.int/en/health-topics/disease-prevention/vaccines-and-immunization/publications/2014/european-vaccine-action-plan-20152020-2014

[5] Musa S, Topalović B, Ćatić S, Smajlagić Z. Assessment of vaccine effectiveness during measles outbreak in the Federation of Bosnia and Herzegovina, 2014-2015. Cent Eur J Public Health. 2018;26:79–82. doi:10.21101/cejph.a4754. 30102493

[6] World Health Organization Regional Office for Europe. 32nd meeting of the European regional certification commission for poliomyelitis eradication. Copenhagen (Denmark): World Health Organization; 2018 [accessed 2019 1127]. http://www.euro.who.int/en/health-topics/communicable-diseases/poliomyelitis/publications/2018/32nd-meeting-of-the-european-regional-commission-for-certification-of-poliomyelitis-eradication-rcc-report-2018

[7] World Health Organization Regional Office for Europe. Tailoring immunization programmes to reach underserved groups – the TIP approach. Copenhagen (Denmark): World Health Organization; 2019 [accessed 2019 1126]. http://www.euro.who.int/tip

[8] Dube E, Leask J, Wolff B, Hickler B, Balaban V, Hosein E, Habersaat K. The WHO Tailoring Immunization Programmes (TIP) approach: review of implementation to date. Vaccine. 2017;36:1509–15. doi:10.1016/j.vaccine.2017.12.012. 29287678 PMC10520948

[9] Bach Habersaat K, Jackson C. Understanding vaccine acceptance and demand—and ways to increase them. Bundesgesundheitsbl. 2020;63:32–39. doi:10.1007/s00103-019-03063-0. PMC6925076 31802154

[10] Michie S, Atkins L, West R. The Behaviour Change Wheel. A Guide to Designing Interventions. Bream (UK): Silverback Publishing; 2014.

[11] Musa S, Skrijelj V, Kulo A, Bach Habersaat K, Smjecanin M, Primorac E, Becirovic D, Jackson C. Identifying barriers and drivers to vaccination: a qualitative interview study with health workers in the Federation of Bosnia and Herzegovina. Vaccine. 2020;38:1906–14. doi:10.1016/j.vaccine.2020.01.025. 31980190

[12] Amirthalingam G, Gupta S, Campbell H. Pertussis immunisation and control in England and Wales, 1957 to 2012: a historical review. Euro Surveillance. 2013;18:20587. doi:10.2807/1560-7917.es2013.18.38.20587. 24084340

[13] World Health Organization. Vaccination coverage cluster surveys: reference manual, Version 3. [accessed 2019 530]. https://www.who.int/immunization/monitoring_surveillance/Vaccination_coverage_cluster_survey_with_annexes.pdf

[14] Bodin J. and Cassel C. Advice on master sample for Bosnia and Herzegovina (BiH), Part II; Report from a mission to BiH. Statistics Sweden, International Consulting Office; 2000.

[15] Institute for statistics of FBiH. Statistical yearbook. Sarajevo; 2016.

[16] Institute for statistics of FBiH. Statistical yearbook. Sarajevo; 2017.

[17] World Health Organization. Measles – european Region. Disease outbreak news – update. Copenhagen (Denmark): World Health Organization; 2019 [accessed 2019 812]. https://www.who.int/csr/don/06-may-2019-measles-euro/en/

[18] World Health Organization/UNICEF. Estimates of National Immunization Coverage (WUENIC). Geneva (Switzerland): World Health Organization; 2018 [accessed 2019 1126]. https://www.who.int/immunization/monitoring_surveillance/data/en/

[19] UNICEF. Knowledge, Attitudes and Practices in Relation to Immunization of Children in Serbia. 2018 [accessed 2019 1121]. https://www.unicef.org/serbia/media/9146/file/Knowledge,%20attitudes%20and%20practices.pdf

[20] Radovanovic Z. Anti-vaccinationists and their arguments in the Balkan countries that share the same language. Srp Arh Celok Lek. 2017;145:46. doi:10.2298/SARH161214046R.

[21] Allan N, Harden J. Parental decision-making in uptake of the MMR vaccination: a systematic review of qualitative literature. J. Public Health. 2015;37:678–87. doi:10.1093/pubmed/fdu075. 25297657

[22] Herzog R, Alvarez-Pasquin MJ, Diaz C, Del-Barrio JL, Estrada JM, Gil A. Are healthcare workers’ intentions to vaccinate related to their knowledge, beliefs and attitudes? A systematic review. BMC Public Health. 2019;13:154. doi:10.1186/1471-2458-13-154. PMC3602084 23421987

[23] Forshaw J, Gerver SM, Gill M, Cooper E, Manikam L, Ward H. The global effect of maternal education on complete childhood vaccination: a systematic review and meta-analysis. BMC Infect Dis. 2017;17:801. doi:10.1186/s12879-017-2890-y. 29281990 PMC5745980

[24] Gowda C, Dempsey AF. The rise (and fall?) of parental vaccine hesitancy. Hum Vacc Immunother. 2013;9:1755–62. doi:10.4161/hv.25085. PMC3906278 23744504

[25] UNICEF. Bosnia and Herzegovina Multiple Indicator Cluster Survey 2011-2012. 2014 [accessed 1812 2019]. https://mics-surveys-prod.s3.amazonaws.com/MICS4/Europe%20and%20Central%20Asia/Bosnia%20and%20Herzegovina/2011-2012/Final/Bosnia%20and%20Herzegovina%202011-12%20MICS_English.pdf

[26] Fournet N, Mollema L, Ruijs WL, Harmsen IA, Keck F, Durand JY, Cunha MP, Wamsiedel M, Reis R, French J, et al. Under-vaccinated groups in Europe and their beliefs, attitudes and reasons for non-vaccination; two systematic reviews. BMC Pub Health. 2018;18:196. doi:10.1186/s12889-018-5103-8. 29378545 PMC5789742

[27] Cook B, Wayne GF, Valentine A, Lessios A, Yeh E. Revisiting the evidence on health and health care disparities among the Roma: a systematic review 2003–2012. Int J Public Health. 2013;58:885–911. doi:10.1007/s00038-013-0518-6. 24096986

